# Alpha 2-macroglobulin acts as a clearance factor in the lysosomal degradation of extracellular misfolded proteins

**DOI:** 10.1038/s41598-023-31104-x

**Published:** 2023-03-28

**Authors:** Ayaka Tomihari, Mako Kiyota, Akira Matsuura, Eisuke Itakura

**Affiliations:** 1 Department of Biology, Graduate School of Science and Engineering, Chiba University, Inage-Ku, Chiba, 263-8522 Japan; 2 Department of Biology, Graduate School of Science, Chiba University, Inage-Ku, Chiba, 263-8522 Japan

**Keywords:** Chaperones, Protein quality control

## Abstract

Proteostasis regulates protein folding and degradation; its maintenance is essential for resistance to stress and aging. The loss of proteostasis is associated with many age-related diseases. Within the cell, molecular chaperones facilitate the refolding of misfolded proteins into their bioactive forms, thus preventing undesirable interactions and aggregation. Although the mechanisms of intracellular protein degradation pathways for intracellular misfolded proteins have been extensively studied, the protein degradation pathway for extracellular proteins remain poorly understood. In this study, we identified several misfolded proteins that are substrates for alpha 2-macroglobulin (α_2_M), an extracellular chaperone. We also established a lysosomal internalization assay for α_2_M, which revealed that α_2_M mediates the lysosomal degradation of extracellular misfolded proteins. Comparative analyses of α_2_M and clusterin, another extracellular chaperone, indicated that α_2_M preferentially targets aggregation-prone proteins. Thus, we present the degradation pathway of α2M, which interacts with aggregation-prone proteins for lysosomal degradation via selective internalization.

## Introduction

Protein misfolding can occur upon exposure to stresses such as heat, oxidation, and pH change. The exposure of hydrophobic sites on misfolded proteins causes non-specific interactions and induces the formation of protein aggregates^1^. Protein aggregates are associated with the onset of neurodegenerative diseases such as Alzheimer's disease^2^; the maintenance of proteostasis prevents loss of function and eliminates toxicity. In mammals, the mechanisms that maintain intracellular proteostasis are well-known. Molecular chaperones interact with misfolded proteins in cells, maintain solubility, and mediate refolding to prevent the aggregation of such proteins^3^. Major molecular chaperones (e.g., heat shock proteins) are upregulated and inhibit protein denaturation when cells are exposed to heat stress^4,5^. Intracellular protein degradation pathways are also essential for proteostasis. The proteasome degrades misfolded proteins that are ubiquitinated by specific E3 ubiquitin ligases^6^. Autophagy degrades damaged proteins and organelles via lysosomal degradation^7^. However, intracellular proteostasis cannot occur outside of the cell; moreover, intracellular chaperones could not mediate refolding if they were present in the extracellular environment because there is a considerably lower concentration of adenosine triphosphate (ATP) in extracellular fluids^3,8^. In addition, extracellular conditions are generally harsher than intracellular conditions^9^. Therefore, a distinct mechanism mediated by extracellular chaperones is required to maintain extracellular proteostasis and prevent aggregation in the extracellular environment. In the first step, an extracellular chaperone interacts with a substrate in the extracellular environment, ensuring the solubility of the substrate. In the second step, the extracellular chaperone–substrate complex is selectively internalized into the cell via endocytosis; it then undergoes lysosomal degradation^10^.

Extracellular chaperones including clusterin, Haptoglobin, alpha 2-macroglobulin (α_2_M) and casein prevent aggregate formation in the extracellular environment^9,11–16^. Clusterin has been characterized in terms of its chaperone activity and its role in the degradation pathway^10,17,18^; however, other extracellular chaperones have not been well characterized, and the degradation pathways of extracellular chaperones remain poorly understood. A clear understanding of the mechanisms by which extracellular chaperones mediate extracellular protein degradation will contribute to the development of novel therapies for various diseases that involve protein degeneration; such diseases include Alzheimer's disease^19^, which impacts approximately 50 million patients worldwide but has no effective treatment. We recently reported that a clusterin–misfolded protein complex is selectively delivered into cells through a cell surface receptor for lysosomal degradation^10^. α_2_M has also been shown to interact with stressed proteins and has been proposed to facilitate their disposal via receptors^20^; however, the degradation pathway of α_2_M remains uncharacterized. In the present study, we investigated α_2_M with the aim of determining its substrate specificity and its role in the lysosomal degradation of extracellular misfolded proteins.α_2_M is a 180-kDa protein that forms a homotetramer^21^; it is mainly produced in the liver and secreted into the blood^22^. α_2_M is the sixth most abundant plasma protein^23^ (2.1 mg/mL^24^); it is also present in cerebrospinal fluid at low levels (1.0–3.6 μg/mL^25^). The plasma concentration of α_2_M increases during inflammatory responses in mice^26^. The molecular mechanism underlying the protease inhibitor effects of α_2_M has been widely studied^21^. When the highly protease-sensitive bait region of α_2_M is cleaved by a protease, a structural change entraps the protease, thus inhibiting protease activity^21^. This change also exposes a receptor recognition site for low-density lipoprotein receptor-related protein (LRP1), which targets the α_2_M–protease complex for endocytic degradation^20,27,28^. Through this mechanism, α_2_M eliminates all classes of proteases from the extracellular environment^29^. α_2_M reportedly exhibits chaperone-like activity and inhibits the aggregation of heat-stressed proteins^20^. However, it remains unclear whether α_2_M participates in the lysosomal degradation of extracellular misfolded proteins. Therefore, in this study, we established a fluorescence internalization assay to measure α_2_M-mediated lysosomal degradation. Using green (GFP) and red fluorescence protein (RFP), the internalization assay quantitatively detects α_2_M on the plasma membrane and in lysosomes. We then conducted a comprehensive analysis to identify blood cell proteins that bind to α_2_M in a misfolding-dependent manner. The results of this study will provide a conceptual framework to explain how extracellular chaperones cooperatively contribute to extracellular proteostasis in the process that leads to lysosomal degradation of misfolded extracellular proteins.

## Results

### α_2_M and misfolded proteins are internalized into cells for lysosomal degradation

**Figure 1:**
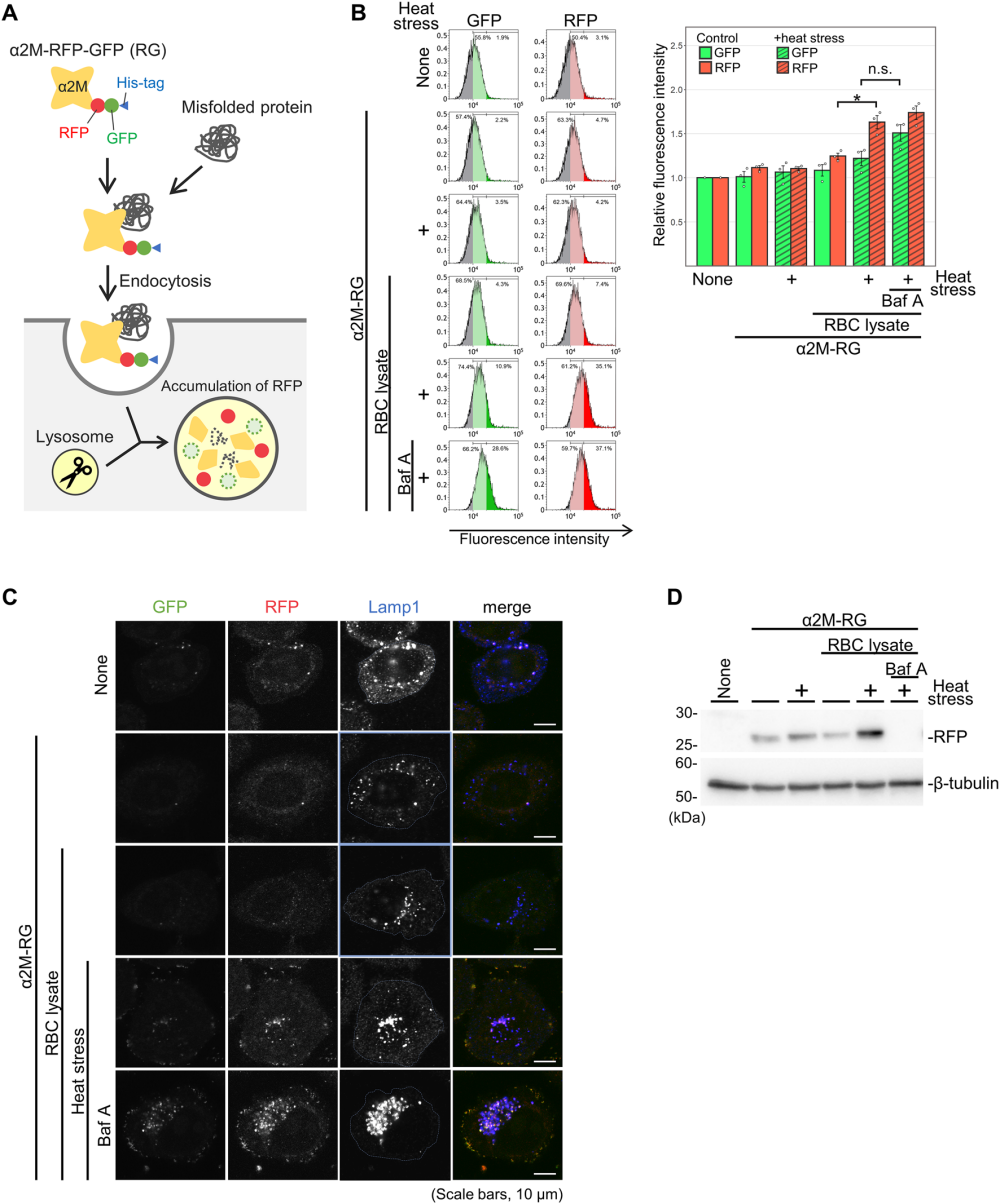
α_2_M induces lysosomal degradation of extracellular misfolded proteins. (**A**) Schematic representation of lysosomal degradation of α_2_M–RFP–GFP–His (–RG). Recombinant α_2_M–RG are internalized into lysosomes, leading to the accumulation of protease- and pH-resistant red fluorescent protein (RFP), but not α_2_M and green fluorescent protein (GFP). If α2M induces degradation of misfolded proteins, RFP should accumulate in the cell only in the presence of misfolded proteins. (**B**) α_2_M–RG internalization assay with red blood cell (RBC) lysate. RBC lysate and α_2_M–RG were heat-shocked in serum-free medium at 50 °C for 1 h. HeLa cells were cultured in the medium for 17 h at 37 °C, then analyzed using flow cytometry. Bar graph shows the relative fluorescence intensities of GFP and RFP in cells normalized to those intensities in untreated cells (n = 3). Data are means ± standard errors of the mean (SEMs). n.s., not significant; *P < 0.05 (two-tailed Student’s *t*-test). Small circles indicate each data point. (**C**) α_2_M–RG internalized into lysosomes. Cells were treated as described in (**B**), immunolabeled with the lysosomal marker LAMP1, and imaged using confocal microscopy. Dashed lines represent cell surface region. Scale bar, 10 μm. (**D**) RFP cleavage assay with α_2_M–RG and RBC lysate. Huh7 cells were treated as described in (**B**), and the cells were lysed for immunoblotting.

We recently discovered that clusterin mediates the lysosomal degradation of extracellular abnormal proteins^10^. To determine whether another extracellular chaperone, α_2_M, also participates in the lysosomal degradation pathway, we produced a recombinant protein of α_2_M fused with two fluorescent proteins (mCherry [an RFP] and superfolder GFP) and a His-tag sequence from mammalian cells. RFP is highly resistant to lysosomal proteases and is pH-insensitive, whereas GFP and α_2_M are not resistant to lysosomal proteases and acidic environments^30^. Therefore, when α_2_M–RFP–GFP–His protein is internalized into lysosomes, the fluorescence of RFP, but not GFP, is detected^10^ (Fig. 1A). To avoid potential structural interference of RFP–GFP (RG) tagging to α_2_M, we prepared two plasmids that expressed α_2_M–RG (–His) or (His–) RG–α_2_M. These plasmids were introduced into Flp-in T-Rex HEK293 cells using the FLP/FRT recombination system to generate stable cell lines. Immunoblotting demonstrated that α_2_M–RG was secreted in the culture supernatant, whereas RG–α_2_M was not (Fig. S1A), which indicated that α_2_M–RG–His was correctly folded. Therefore, we purified α_2_M–RG protein from the conditioned medium (Fig. S1B). We characterized the conformational state of α_2_M-RG using Native-polyacrylamide gel electrophoresis (Native-PAGE) and methylamine, which undergoes conformational collapse of α_2_M that is highly similar to its protease-cleaved conformation by　aminolysis of its thiol ester^31,32^. Native-PAGE revealed that α_2_M–RG migrated to ~ 920 kDa, and treatment of methylamine results in faster migration of α_2_M–RG (collapsed a2M-RG) than untreated α_2_M–RG (Fig. S1C). An intact thiol ester of α_2_M apparent from formation of characteristic heat-induced autolysis products in sodium dodecyl sulfate (SDS)-PAGE^31^. Purified α_2_M–RG was boiled in SDS sample buffer and analyzed by SDS-PAGE. The result showed that the thiol-ester-dependent heat-fragmentation bands (TE120 for N-terminus and TE110 for C-terminus with RG) were generated, which were inhibited by methylamine (Fig. S1D). These results indicate that purified α_2_M–RG is a tetramer with an intact thiol ester.

Haptoglobin captures hemoglobin leaking into plasma from intravascular hemolysis; this leads to lysosomal degradation of the hemoglobin. However, scavenger systems for other hemolysis-related intracellular proteins remain unclear. Therefore, we used red blood cell (RBC) lysate as a model substrate for α_2_M. To investigate whether α_2_M–RG is involved in the degradation of denatured proteins, we performed an α_2_M–RG internalization assay. RBC lysate and recombinant α_2_M–RG were heat-shocked in culture in advanced Dulbecco’s Modified Eagle Medium (DMEM) without serum at 50 °C for 1 h. HeLa cells were cultured in the resultant α_2_M–RG-containing media for 17 h at 37 °C. After incubation, the cells were collected; the fluorescence intensities of GFP and RFP were measured by flow cytometry (Fig. 1B). Incubation with α_2_M–RG alone or with RBC lysate did not strongly affect the RFP signal in the cells. However, heat-stressed lysate induced a statistically significant 1.3-fold increase in the RFP signal, compared with non-heat stress conditions. The fluorescence intensity of GFP tended to increase in cells treated with the lysosomal inhibitor bafilomycin A_1_ (Baf A), suggesting that α_2_M–RG is internalized into cells in the presence of misfolded proteins.

To observe the subcellular localization of α_2_M–RG, HeLa cells were subjected to identical treatment, then immunostained with an anti-LAMP1 antibody as a lysosomal marker (Fig. 1C). The number and intensity of RFP dots were elevated in the presence of heat-stressed RBC lysate; RFP dots were co-localized with LAMP1, indicating that α_2_M–RG was internalized into the lysosome. Importantly, treatment with Baf A enhanced RFP and GFP signals on both lysosomes and the cell membrane, suggesting that secondary inhibition of endocytosis by Baf A causes α_2_M–RG accumulation on the cell surface; this accumulated α_2_M–RG may be bound to an unknown cell surface receptor. Next, we confirmed lysosomal degradation of α_2_M–RG using an RFP cleavage assay^33^, which examines the release of free 25-kDa RFP that originates from the digestion of 250-kDa α_2_M–RG by lysosomal proteases. Free RFP was detected in cells treated with α_2_M–RG; the amount of free RFP was further increased in the presence of heat-stressed RBC lysate (Fig. 1D). Baf A inhibited the increase of free RFP. These cell biological and biochemical data demonstrate that α_2_M–RG in complexes with denatured RBC proteins is subjected to lysosomal degradation.

### Identification of heat stress-dependent α_2_M-binding proteins

**Figure 2:**
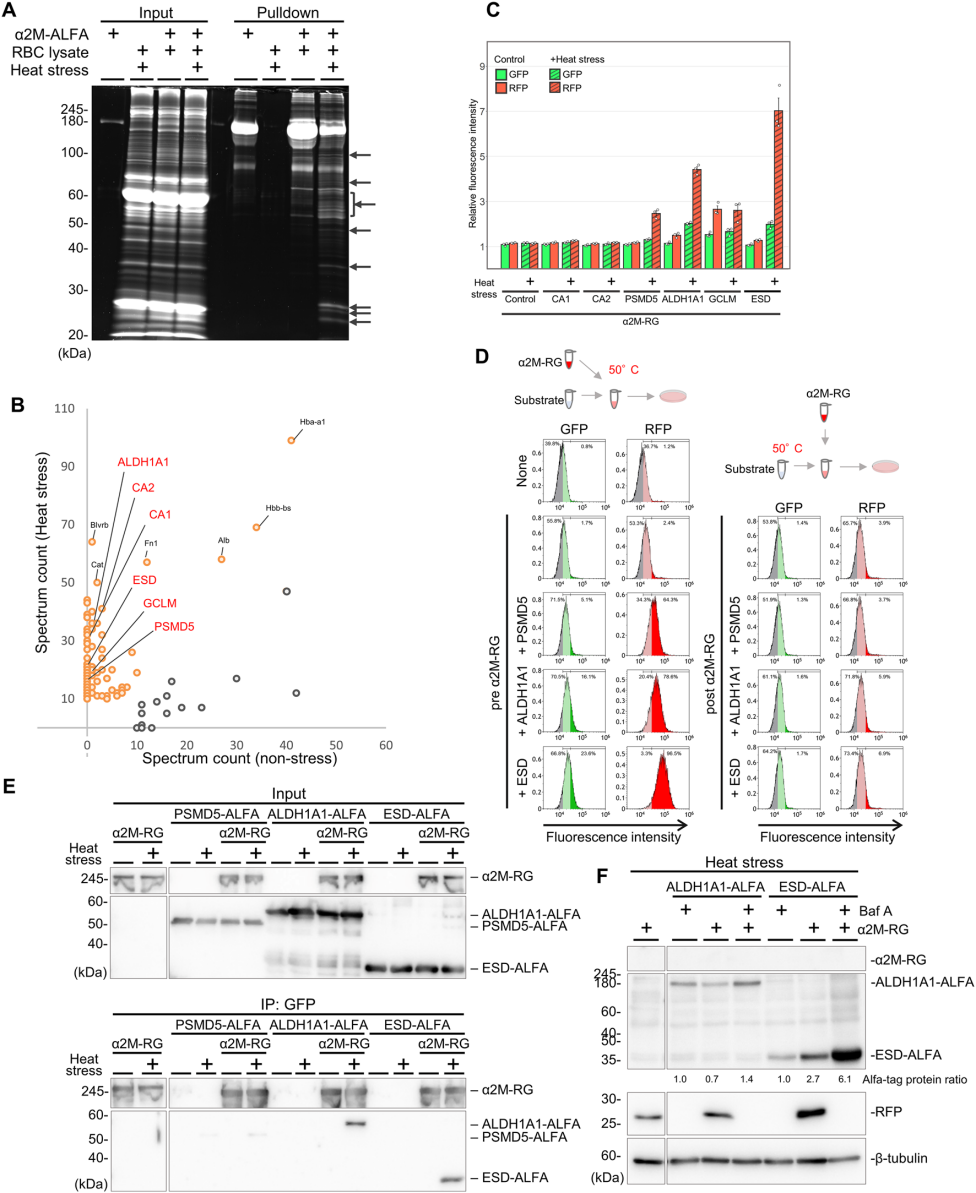
Identification of misfolding-dependent substrates for α_2_M. (**A**) Several heat-stressed proteins directly interacted with α_2_M. Purified α_2_M–ALFA was mixed with or without RBC lysate, then pre-incubated at 4 °C or 50 °C (heat stress) for 1 h. Samples were subjected to co-immunoprecipitation with anti-ALFA sepharose and analyzed by ruby staining. (**B**) Mass spectrometric analysis of α_2_M-binding proteins (see also Table S1). Orange (gray) dots indicate proteins that bind α_2_M in a heat stress-dependent (independent) manner. (**C**) α_2_M–RG internalization assay with substrates. Six substrates (CA1, CA2, PSMD5, ALDH1A1, GCLM, and ESD) and α_2_M–RG were heat-shocked in serum-free medium at 50 °C for 1 h. HeLa cells were cultured in medium for 17 h at 37 °C, then analyzed using flow cytometry. Bar graph shows the relative fluorescence intensities of GFP and RFP in cells normalized to those intensities in untreated cells (n = 3). Data are means ± SEMs. Small circles indicate each data point. (**D**) α_2_M did not deliver aggregated proteins to lysosomes. Substrates (PSMD5, ALDH1A1, or ESD) were co-heated with α_2_M–RG at 50 °C (left panel) or pre-heated alone, then mixed with α_2_M–RG (right panel). HeLa cells were cultured in medium for 17 h at 37 °C, then analyzed using flow cytometry (n = 1). (**E**) α_2_M interacted directly with substrates. PSMD5, ALDH1A1, and ESD were each mixed with or without α_2_M–RG, then pre-incubated at 4° or 50 °C (heat stress) for 1 h. Samples were subjected to co-immunoprecipitation with anti-GFP sepharose. (**F**) α_2_M–RG cleavage assay with ALDH1A1 and ESD. Huh7 cells were treated as described in (**C**), and the cells were lysed for immunoblotting using anti-GFP, anti-ALFA RFP, and anti-β-tubulin. Quantitative analysis of band intensity using ImageJ. Data are presented as the ratio of Alfa-tag protein (α_2_M–RG or α_2_M–RG + Baf A treated sample/Baf A treated sample) after normalization to tubulin as mean of two independent experiments.

To identify RBC proteins that bound to α_2_M–RG in a misfolding-dependent manner, we performed co-immunoprecipitation using α_2_M fused to an ALFA-tag, a 13-amino acid tag that forms a stable α-helix^34^. A mixture of recombinant α_2_M–ALFA and RBC lysate was heated at 50 °C for 1 h, then subjected to co-immunoprecipitation. Interactions of α_2_M–RG with several proteins under heat stress were observed (Fig. 2A); we identified 142 proteins via mass spectrometry (Table S1). Spectra from the heat-stressed sample and non-heat-stressed control were graphically compared (Fig. 2B). We selected 12 candidate proteins for evaluation in pilot experiments (α_2_M–RG and clusterin–RG internalization assay with purified recombinant proteins), then extracted six proteins as candidate substrates for α_2_M: carbonic anhydrase 1 (CA1), carbonic anhydrase 2 (CA2), 26S proteasome non-ATPase regulatory subunit 5 (PSMD5), retinal dehydrogenase 1 (ALDH1A1), glutamate–cysteine ligase regulatory subunit (GCLM), and S-formylglutathione hydrolase (ESD). After purification of these recombinant proteins, including CA1, CA2, PSMD5, ALDH1A1, GCLM, and ESD, from E. *coli* (Fig. S2), an α_2_M–RG internalization assay using the purified recombinant proteins showed that the fluorescence intensity of RFP significantly increased in the presence of heat-stressed PSMD5, ALDH1A1, GCLM, and ESD (Fig. 2C). However, GCLM increased the fluorescence intensity of RFP regardless of heat stress, suggesting that its ability to bind α_2_M may be non-specific or different functions. In contrast, we detected no increase in the fluorescence intensity of RFP in an RFP–GFP internalization assay using the recombinant proteins (Fig. S3A); this finding indicated that α_2_M mediates internalization. α_2_M–RG internalization assays with CA1 and CA2 did not show changes in the fluorescence intensity of RFP. To determine whether α_2_M recognizes misfolded or aggregated proteins, substrate (PSMD5, ALDH1A1, or ESD) and α_2_M–RG were co-heated at 50 °C (Fig. 2D, left panel), substrates were pre-heated alone and then mixed with α_2_M–RG (Fig. 2D, right panel), or α_2_M–RG or substrate was pre-heated alone and then mixed with together (Fig. S3B); all mixtures were analyzed using an internalization assay. Flow cytometry analysis showed that pre-heated substrates or pre-heated α_2_M–RG did not induce α_2_M–RG internalization. Co-immunoprecipitation using recombinant proteins demonstrated that α_2_M–RG directly interacted with PSMD5, ALDH1A1, and ESD in a heat stress-dependent manner (Fig. 2E). Next, we explored whether the substrates were internalized into lysosomes by α_2_M–RG. Immunoblotting analysis confirmed that the amount of intracellular ESD increased in the presence of α_2_M–RG (Fig. 2F). Co-treatment of α_2_M–RG with the lysosomal inhibitor Baf A led to ALDH1A1 and ESD accumulation. These data indicate that α_2_M directly interacts with misfolded proteins and leads to the lysosomal degradation of the substrate.

### Lysosomal degradation of the α_2_M–misfolded protein complex is a universal system present in various tissue-derived cells

**Figure 3:**
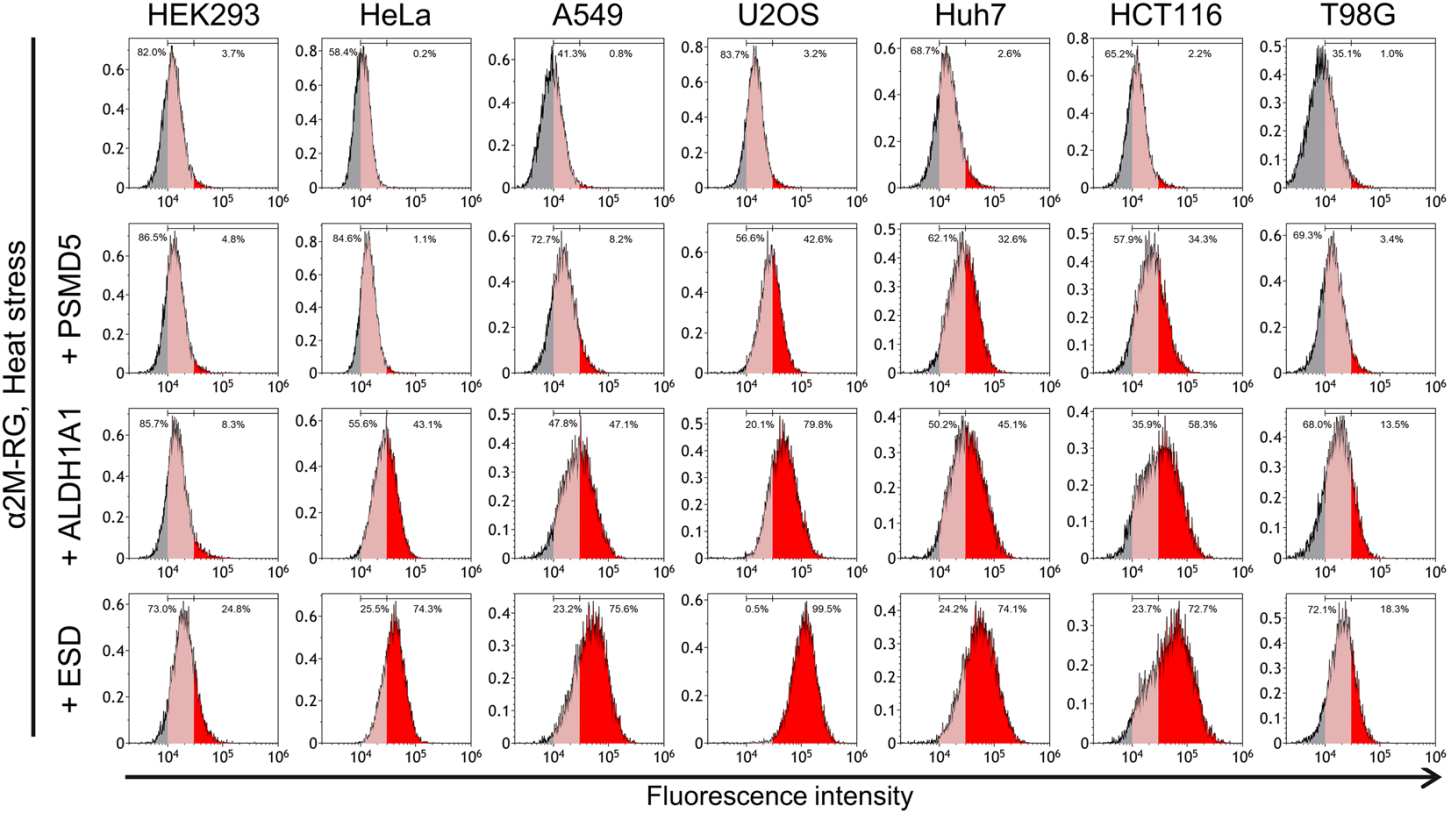
Lysosomal degradation of α_2_M with substrate is ubiquitous in different cell lines. α_2_M–RG internalization assay with PSMD5, ALDH1A1, or ESD. In the absence or presence of substrate, α_2_M–RG was heat-shocked in serum-free medium at 50 °C for 1 h. Each cell line (HEK293, HeLa, A549, U2OS, Huh7, HCT116, or T98G) was cultured in medium for 17 h at 37 °C, then analyzed using flow cytometry (n = 1).

We conducted α_2_M–RG internalization assays using seven cell lines derived from different origins: HEK293 (embryonic kidney), HeLa (cervical cancer), A549 (lung cancer), U2OS (osteosarcoma), Huh7 (liver carcinoma), HCT116 (colon cancer), and T98G (glioblastoma). Most cell lines showed some degree of RFP signal enhancement in the presence of heat-stressed proteins (Fig. 3), suggesting that the α_2_M complex-degrading mechanism is a ubiquitous system.

### The bait region promotes α_2_M internalization

**Figure 4:**
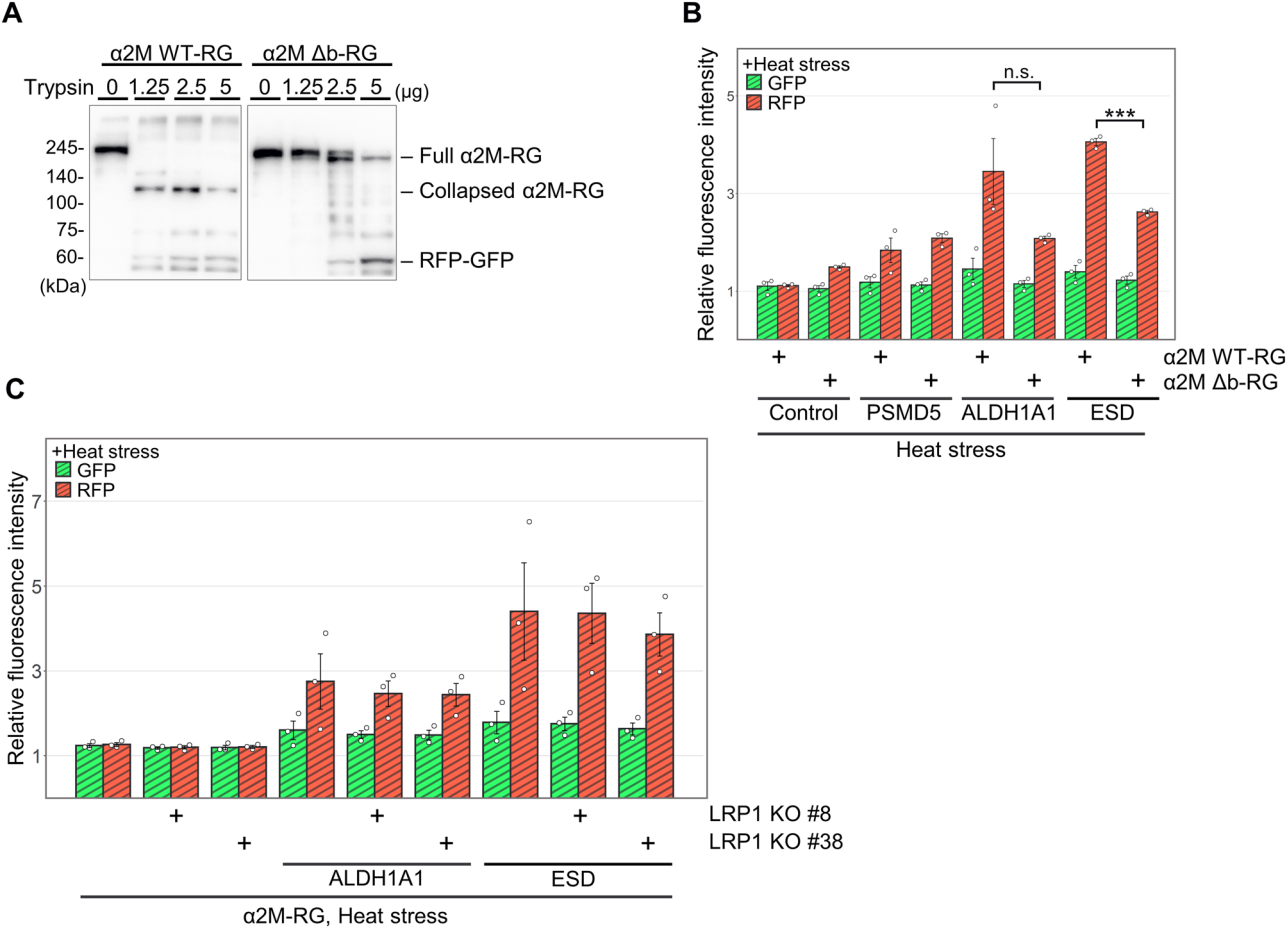
The bait region of α_2_M promotes lysosomal degradation. (**A**) The bait region of α_2_M Δb–RG is not cleaved by trypsin. Recombinant α_2_M WT–RG or α_2_M Δb–RG was treated with trypsin at 4 °C for 30 min, then analyzed by immunoblotting using anti-GFP. (**B**) α_2_M Δb–RG and α_2_M WT–RG internalization assays with substrates. Mixtures of substrate (PSMD5, ALDH1A1, or ESD) and α_2_M–RG (WT or Δb) were heat-shocked in serum-free medium at 50 °C for 1 h. HeLa cells were cultured in medium for 17 h at 37 °C, then analyzed using flow cytometry. Bar graph shows the relative fluorescence intensities of GFP and RFP in cells normalized to those intensities in untreated cells (n = 3). Data are means ± SEMs. n.s., not significant; ***P < 0.005 (two-tailed Student’s *t*-test). Small circles indicate each data point. (**C**) α_2_M–RG internalization assay with LRP1 KO cells. Substrate (ALDH1A1 or ESD) and α_2_M–RG were heat-shocked in serum-free medium at 50 °C for 1 h. HeLa WT and LRP1 KO cells were cultured in medium for 17 h at 37 °C, then analyzed using flow cytometry. Bar graph shows the relative fluorescence intensities of GFP and RFP in cells normalized to those intensities in untreated cells (n = 3). Data are means ± SEMs. n.s., not significant; ***P < 0.005 (two-tailed Student’s *t*-test). Small circles indicate each data point. KO, knockout; WT, wild type.

α_2_M sequesters proteases through a conformational change triggered by cleavage of the bait region, an unstructured 39-amino acid sequence. To investigate the involvement of the bait region in α_2_M–misfolded protein internalization, we generated α_2_M Δb–RG, a mutant in which the bait region is entirely replaced with a glycine–serine linker sequence that is not susceptible to protease cleavage^31^. The purified α_2_M wild type (WT) and Δb were incubated with trypsin to assess cleavage of the bait region. We detected a 120-kDa fragment of α_2_M–RG, which is a protease-cleaved conformation (collapsed α_2_M), in the α_2_M WT–RG condition, whereas no 120-kDa fragment was produced in the α_2_M Δb–RG condition (Fig. 4A). Trypsin treatment at a higher concentration resulted in random degradation. Thus, the protease-sensitive bait region of α_2_M Δb–RG was completely abolished.

α_2_M Δb–RG internalization assays showed that the increase in RFP intensity after treatment with α_2_M Δb–RG and ESD was 0.65-fold lower than the increase after treatment with α_2_M WT–RG (Fig. 4B). Conversely, there was a marginal difference after treatment with ALDH1A1; no effect was observed after treatment with PSMD5. These results suggest that the bait region is not essential for α_2_M internalization, although it promotes cellular uptake for specific substrates.

Because LRP1 is a cell-surface receptor for the α_2_M–protease complex^35,36^, we explored whether LRP1 mediates internalization of the α_2_M–misfolded protein complex. LRP1 knockout (KO) cell lines were generated using Cas9 and two different gRNAs for LRP1 (gLRP1 #8 or gLRP1 #38); LRP1 expression was completely undetectable in both cell lines (Fig. S4). However, α_2_M–RG in the presence of heated ALDH1A1 and ESD was internalized even in LRP1 KO cells; this finding suggested that LRP1 is not involved in the α_2_M–misfolded protein degradation pathway (Fig. 4C).

### α_2_M and clusterin have distinct specificities as extracellular chaperones

**Figure 5:**
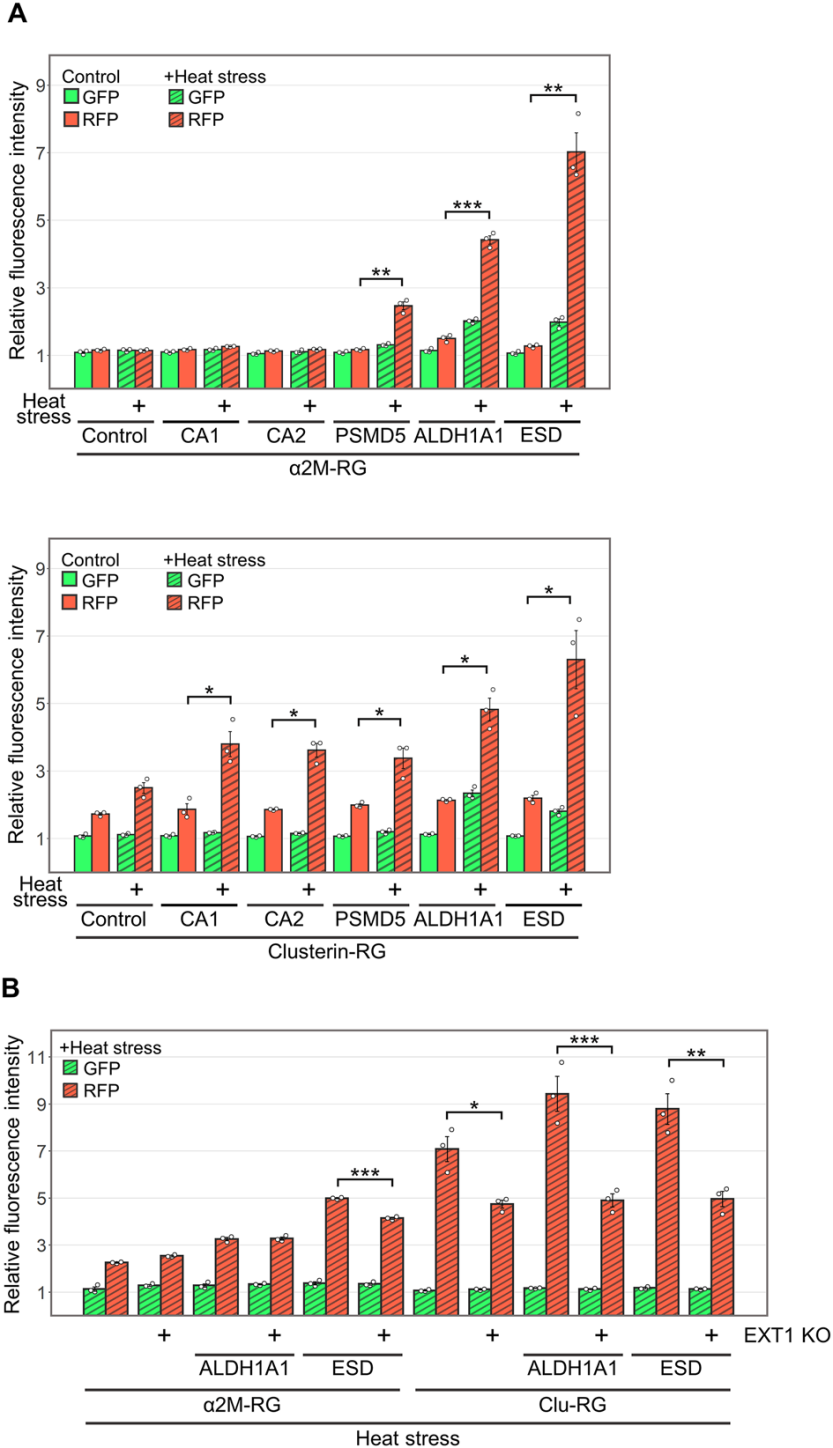
Degradation activity of α_2_M differed from degradation activity of clusterin. (**A**) α_2_M–RG and Clu-RG internalization assays. Substrates (CA1, CA2, PSMD5, ALDH1A1, and ESD) were each mixed with α_2_M–RG or Clu-RG; mixtures were heat-shocked in serum-free medium at 50 °C for 1 h. HeLa cells were cultured in medium for 17 h at 37 °C, then analyzed using flow cytometry. (**B**) α_2_M–RG and Clu-RG internalization assays with EXT1 KO cells. Substrates (ALDH1A1 and ESD) were each mixed with α_2_M–RG or Clu-RG; mixtures were heat-shocked in serum-free medium at 50 °C for 1 h. HeLa WT and EXT1 KO cells were cultured in medium for 17 h at 37 °C, then analyzed using flow cytometry. Bar graph shows the relative fluorescence intensities of GFP and RFP in cells normalized to those intensities in untreated cells (n = 3). Data are means ± SEMs. *P < 0.05; **P < 0.01; ***P < 0.005 (two-tailed Student’s *t*-test). Small circles indicate each data point.

To compare substrate selectivity between α_2_M and clusterin, we used the internalization assay to evaluate the degradation efficiencies of recombinant clusterin–RFP–GFP (Clu-RG) and α_2_M–RG with five substrates. Three substrates (PSMD5, ALDH1A1, and ESD) promoted the lysosomal degradation of both chaperones in a similar manner (Fig. 5A). In contrast, after treatment with Clu-RG, RFP intensity was efficiently elevated twofold by the addition of CA1 or CA2, which did not promote the degradation of α_2_M–RG (Fig. 5A). These results suggest that α_2_M and clusterin have distinct substrate selectivities. Co-immunoprecipitation analysis also showed that binding to clusterin increased in a heat stress-dependent manner for all substrates (Fig. S5).

Because our previous work suggests that the clusterin–misfolded protein complex binds to the heparan sulfate receptor^10^, we explored whether heparan sulfate is required for α_2_M. We performed an internalization assay of α_2_M–RG and Clu-RG using cells that lacked EXT1 (an essential synthase for heparan sulfate; i.e., EXT1 KO cells). As expected, increased uptake of Clu-RG was substantially inhibited in EXT1 KO cells, compared to control cells. In contrast, EXT1 depletion only marginally inhibited the uptake of α_2_M compared with clusterin (Fig. 5B), indicating that α_2_M uses a cell surface receptor different to that used by clusterin.

### α_2_M had an affinity for aggregation-prone proteins

**Figure 6:**
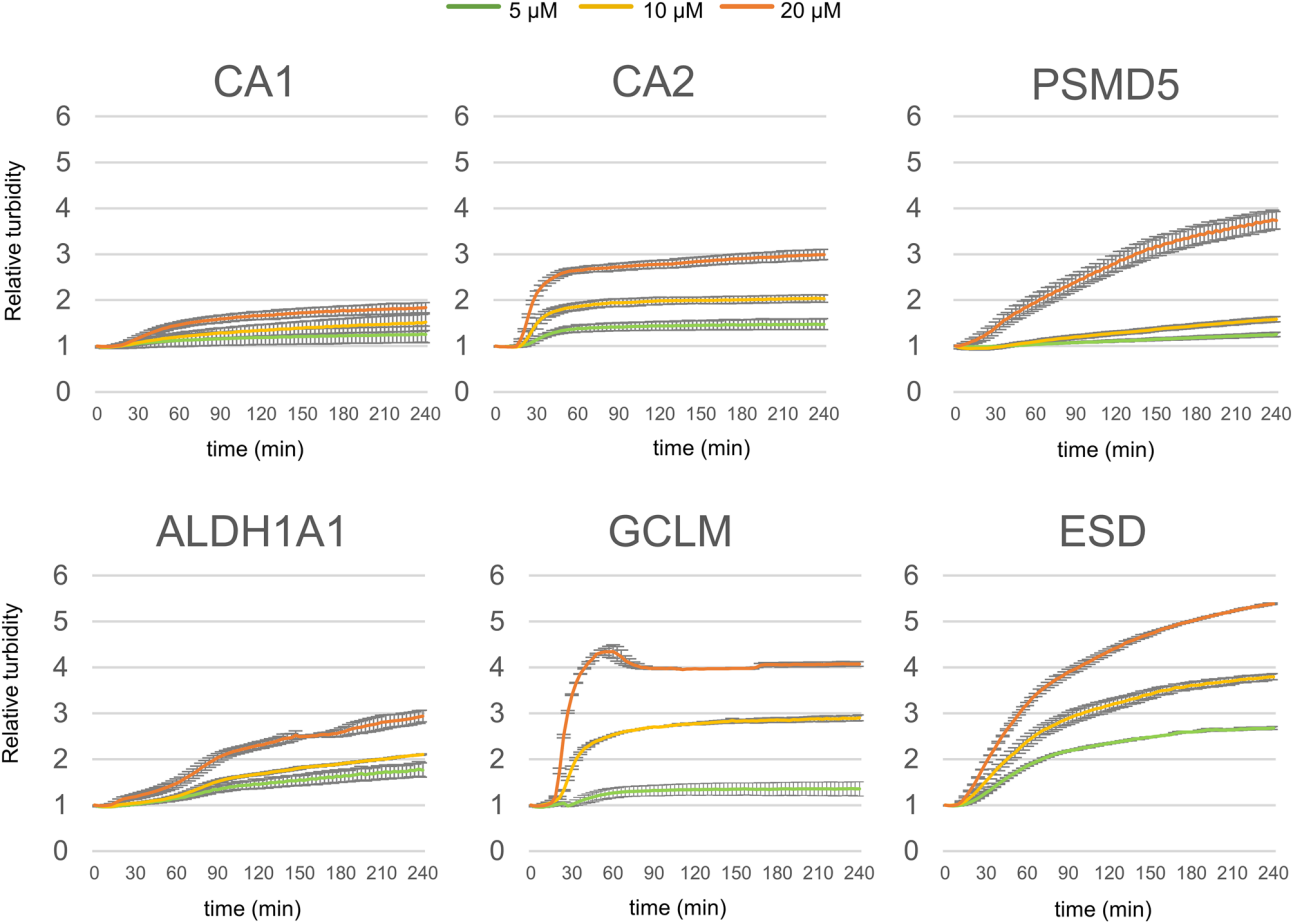
CA1 and CA2 exhibit low and rapid aggregation, respectively. Substrates (CA1, CA2, PSMD5, ALDH1A1, or ESD) were incubated in phosphate-buffered saline (PBS) at 50 °C for 4 h (n = 3). Turbidity (A360) was measured at 3-min intervals at 5 μM (green), 10 μM (yellow), and 20 μM (orange). Relative turbidity is calculated as the ratio of turbidity at each time point to the initial turbidity. Data are means ± SEMs.

CA1 and CA2 underwent efficient clusterin-mediated lysosomal degradation, whereas they did not undergo α_2_M-mediated lysosomal degradation (Fig. 5A). To examine the properties of substrates targeted by α_2_M or clusterin, we performed a turbidity assay at 50 °C and categorized the heating results into three groups. In the first group, heating of CA2, PSMD5, and ALDH1A1 resulted in a large increase in turbidity. Heating of ESD produced the greatest protein precipitation among the five tested substrates (i.e., increased absorbance at 360 nm; Fig. 6A), suggesting that aggregation-prone proteins had strong affinity for both chaperones. In contrast, heating of CA1 led to a small increase in turbidity. In the second group, heating of CA2 led to a rapid increase in turbidity within 60 min, whereas other substrates exhibited progressive increases in turbidity over 240 min. In the third group, heating of CA1, CA2, and PSMD5 revealed a modest formation of aggregates at a low concentration (5 μM). Overall, these results indicate that α_2_M preferentially targets aggregate-prone misfolded proteins that are produced by gradual denaturing, whereas clusterin targets a broad range of denatured proteins.

## Discussion

Extracellular chaperones have been evaluated for their importance in extracellular proteostasis. The chaperone activity of extracellular chaperones against stressed proteins has been characterized relatively well^16,17^. In contrast, less is known about the degradation pathways of extracellular chaperones except that of clusterin^10,18^. In this study, we found that α_2_M mediates lysosomal degradation of extracellular misfolded proteins, thus maintaining extracellular proteostasis. Our data indicate that although the effects of α_2_M overlap with the effects of clusterin, α_2_M recognizes misfolded proteins depending on their level of aggregation. An α_2_M internalization assay showed that α_2_M also mediates the cellular internalization of extracellular misfolded proteins. α_2_M is a protease inhibitor and might inhibits lysosomal proteases after transport into lysosomes. However, lysosomes contain approximately 10 proteases, including serine-, cysteine-, and aspartate-type acidic proteases^37^. α_2_M would be eventually degraded by various lysosomal proteases. Thus, a key function of α_2_M is the removal of misfolded proteins generated by exposure to stress in the harsh extracellular environment.

Thus far, no quantitative method has been available to detect the lysosomal degradation of extracellular proteins; the clearance of stressed protein by α_2_M has been poorly understood. Although a conventional method for the detection of internalization involves fluorescence labeling of extracellular proteins, this method cannot distinguish between cell surface interaction and lysosomal internalization. To overcome this problem, we designed the recombinant protein α_2_M–RG, which is composed of two fluorescent proteins, RFP and GFP. Using α_2_M–RG, we quantitatively detected an increase in RFP, but not GFP, during the cellular internalization of α_2_M–RG. Biochemical and cell biological experiments revealed that α_2_M is involved in the lysosomal degradation of heat-stressed proteins (Fig. 1). Since the RFP-GFP tag is a large protein, it may affect the functions of tagged α2M. However, RFP-GFP does not facilitate the degradation of misfolded proteins (Fig. S2); therefore, facilitation of the degradation of misfolded proteins of α2M–RG is due to α2M.

Mass spectrometry revealed that PSMD5, ALDH1A1, and ESD were α_2_M-interacting proteins in RBC lysate; these proteins formed a complex with α_2_M under heat stress (Fig. 2E) and were internalized (Fig. 2F). Our findings suggest that these substrates are internalized into the cell together with α_2_M, where they undergo lysosomal degradation. Intravascular hemolysis causes autoimmune diseases^38^, infection^39^, and mechanical stress^40^ (e.g., running^41^), which might be associated with accumulation of normally intracellular proteins. Because more than half of all cells in the human body are erythrocytes, sufficient hemolysis would expose numerous intracellular proteins to the extracellular environment. Therefore, RBC-derived intracellular proteins in the blood may produce misfolded proteins in the harsh extracellular environment and might contribute to disease. In addition, α_2_M is abundant in blood^23^, and α_2_M internalization was confirmed in most of the cultured cells tested in this study (Fig. 3). Based on the present findings, we propose that α_2_M protects extracellular proteostasis from the effects of hemolysis.α_2_M functions as a protease inhibitor that traps and inactivates proteases via conformational changes caused by protease-induced cleavage of the bait region^21^. To investigate the relationship between the bait region and lysosomal internalization, we used the bait region mutant α_2_M Δb–RG^31^, in which the bait region was completely replaced with a glycine–serine linker sequence. α_2_M Δb–RG reduced internalization compared to WT (Fig. 4B). Although the bait region was not essential for α_2_M degradation in the presence of misfolded protein, our data suggest that the bait region facilitates degradation. Since the culture medium used during the internalization assay did not contain a protease (we used advanced DMEM/F12 without serum), the bait region might contribute to another role without cleavage. Although a cell surface receptor might be involved in the recognition of α2M–misfolded protein complexes via the bait region, LRP1 was not essential for lysosomal degradation of the α2M–misfolded protein complexes under our conditions (Fig. 4C). LRP1 may have another role, such as mediating α2M recycling via the recycling endosome^42^. For lysosomal degradation of the α2M–misfolded protein complex, other α2M receptors, such as Grp78, might be involved^43^.

**Figure 7:**
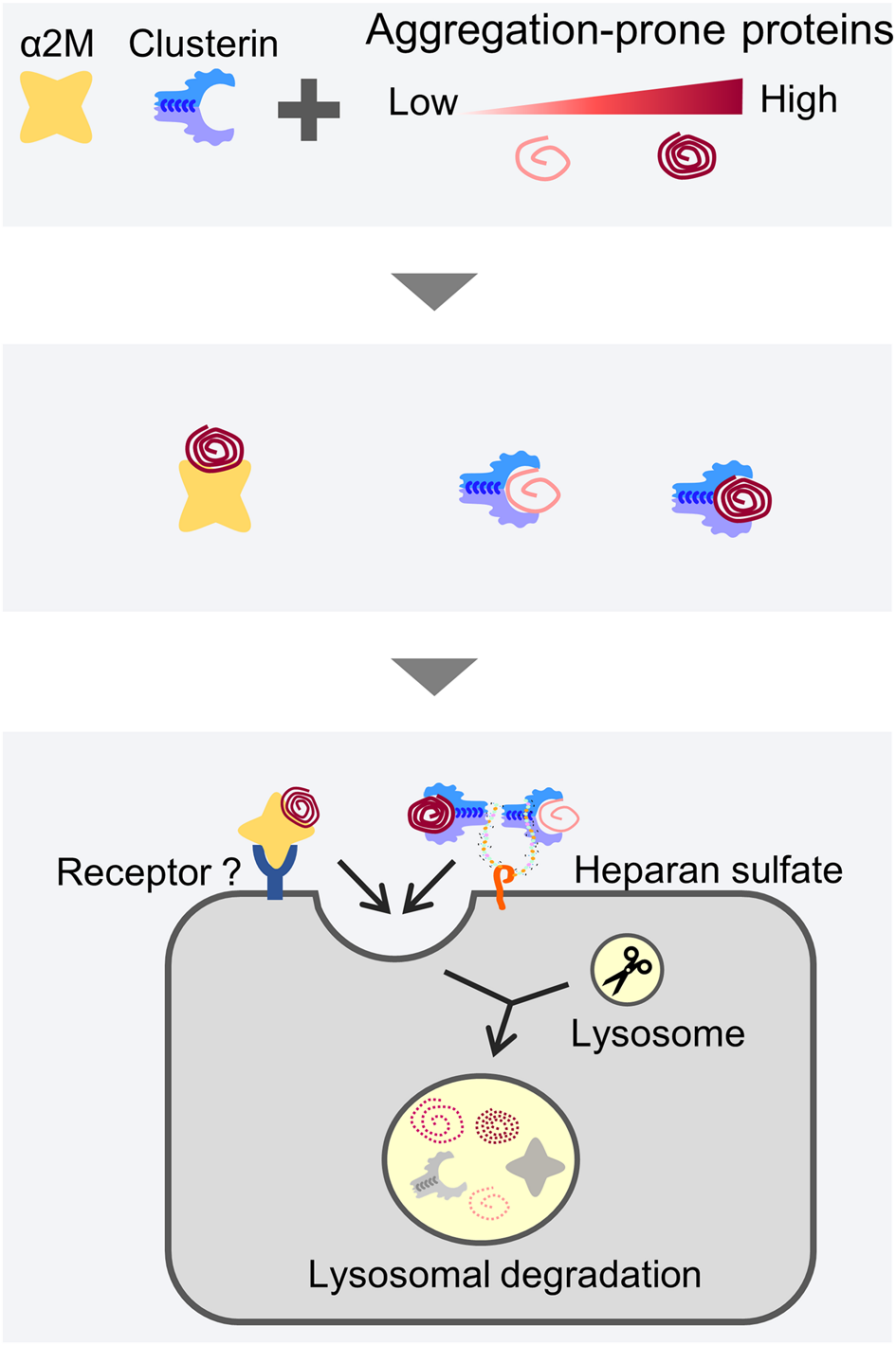
Model of the extracellular misfolded protein degradation pathway by extracellular chaperones. Clusterin targets a broad range of misfolded proteins than α_2_M. The binding kinetics of the extracellular chaperone might be one determinant of the substrate recognition of an extracellular chaperone. The α_2_M–misfolded protein complex is internalized via a putative receptor, leading to degradation of the α_2_M–misfolded protein complex.

To investigate the physiological significance of the presence of multiple extracellular chaperones, we compared the substrate specificities of α_2_M and clusterin. Internalization assays showed that misfolded CA1 increased the degradation of clusterin, but did not affect the degradation of α_2_M, suggesting that α_2_M has comparatively greater substrate selectivity. The turbidity assay revealed that the aggregation growth phase of substrates for α2M and clusterin, including PSMD5, ALDH1A1, and ESD, slowly increased the turbidity (Fig. 6). In contrast, CA1 and CA2, both substrates for clusterin (Fig. 5), quickly reached a plateau. Since α2M did not induce the internalization of aggregated proteins (i.e., preheated proteins) (Fig. 2D), these results suggest that clusterin has higher binding kinetics to misfolding proteins than does α2M. Thus, the binding kinetics and other currently unknown differences in chaperone action may contribute to the different misfolded client protein specificities seen for clusterin and α2M. From this perspective, α_2_M-mediated lysosomal degradation and clusterin-mediated lysosomal degradation are not totally redundant pathways. Therefore, we propose that the cooperation of multiple types of extracellular chaperones is effective for protecting extracellular proteostasis from diverse misfolded proteins in harsh extracellular conditions (Fig. 7).

This study provides evidence that different receptors mediate lysosomal degradation of clusterin or α_2_M-misfolded protein complexes. Because clusterin and α_2_M are both associated with protein deposition diseases such as Alzheimer's disease, prion disease, and atherosclerosis^16,44^, both chaperones have similar physiological roles. On the other hand, clusterin is conserved only among vertebrates^45^, whereas α_2_M is evolutionarily conserved from bacteria to vertebrates^46^, suggesting that α_2_M is a universal factor under diverse conditions. Our internalization assay of α2M–RG suggested cell specificity of the lysosomal degradation of α2M–misfolded protein complexes (Fig. 3). Identifying the tissue specificity of the degradation pathway will provide important information about the quality control system of extracellular proteostasis in vivo.

## Materials and methods

### Plasmids

We amplified α_2_M cDNA from HepG2 total cDNA via polymerase chain reaction and inserted it into a pcDNA5 FRT TO vector along with mCherry, sfGFP, His-tag, and signal sequence (ss) of prolactin (only RG–α_2_M) to generate the fusion proteins α_2_M–mCherry–sfGFP–His (α_2_M–RG) and ss–His–mCherry–sfGFP–α_2_M (RG–α_2_M). For the mutated bait region, the bait region (117 bp) of pcDNA5 FRT TO a2M–RG–His was replaced by a GGS repeat sequence via mutagenesis. For bacterial expression, CA1 from mouse lung total cDNA, CA2 from HEK293 total cDNA, ESD from HEK293 total cDNA, PSMD5 from HeLa total cDNA, ALDH1A1 from HEK293 total cDNA, and GCLM from HEK293 total cDNA were amplified and inserted into a pRSET-A vector along with ALFA tag and His-tag. The resultant plasmids were as follows: pcDNA5 FRT TO α_2_M–mCherry–sfGFP–His, pcDNA5 FRT TO ss–His–mCherry–sfGFP–α_2_M, pcDNA5 FRT TO α_2_M–ALFA–His, pcDNA5 FRT TO α_2_M(Δbait)–mCherry–sfGFP–His, pRSET-A CA1–ALFA–His, pRSET-A CA2–ALFA–His, pRSET-A His–ALFA–3C–PSMD5, pRSET-A His–ALFA–3C–ALDH1A1, pRSET-A His–ALFA–3C–GCLM, and pRSET-A His–ALFA–3C–ESD. pOG44 was used in the Flp-in system. pCMV-VSVG (Addgene plasmid #8454) and psPAX2 (Addgene plasmid #12260) were used for lentivirus production. The pcDNA5 FRT TO FLAG–Cas9 vector was previously described (Itakura et al., 2016). pLenti gEXT1a was previously described (Itakura et al. 2019).

### Antibodies

Rabbit polyclonal anti-LAMP1 antibodies were gifted from Y. Tanaka (Kyushu University, Fukuoka, Japan). Mouse monoclonal anti-GFP (clone no. mFX75, cat no. 012-22541) antibody was purchased from Wako. Mouse monoclonal anti-RFP (clone no. 1G9, cat no. M204-3) antibody was purchased from MBL. Rabbit monoclonal anti-LRP1 (clone no. EPR3724, cat. no. ab92544) antibody was purchased from Abcam. Polyclonal anti-ALFA tag antibody was raised in rabbits by immunization with the ALFA peptide; this antibody generation was performed by Eurofins. GFP-nanobody and ALFA tag-nanobody sepharose were generated by conjugating GFP-nanobody protein purified from pOPINE GFP nanobody (Addgene plasmid #49172) and ALFA tag-nanobody protein^34^ purified from pRSET-A ALFA–His to N-hydroxy succinimide-activated Sepharose 4 Fast Flow (GE).

### Cell culture

Flp-in T-Rex HEK293 (Thermo Fisher Scientific), HeLa (RIKEN BRC), Huh7 (RIKEN BRC), A549 (RIKEN BRC), HCT116 (RIKEN BRC), HEK293FT (Thermo Fisher Scientific), and U2OS cells were cultured in DMEM (Nacalai Tesque); T98G cells were cultured in Eagle’s Minimum Essential Medium (Nacalai Tesque). Each medium was supplemented with 10% fetal bovine serum (Biosera) and 50 µg/mL penicillin/streptomycin (regular medium) in a humidified 5% CO_2_ incubator at 37 °C. Flp-in T-Rex HEK293 cells were maintained in the presence of 100 μg/mL zeocin and 15 μg/mL blasticidin. To generate stable doxycycline (dox)-inducible secreted proteins or FLAG–Cas9-expressing cells, plasmids encoding the respective proteins were co-transfected with pOG44, encoding the FLP recombinase, into Flp-in T-Rex HEK293 cells. Transfected cells were selected by adding 100 μg/mL hygromycin, then maintained in the presence of 15 μg/mL blasticidin and 100 μg/mL hygromycin. Dox (100 ng/mL) was used to induce the integrated gene at the FRT site. Lentivirus-infected HeLa cells harboring Lenti Cas9 Blast were used to generate stable FLAG–Cas9-expressing cells.

### Generation of KO cells using clustered regularly interspaced short palindromic repeats (CRISPR)

SgRNA sequences for KO (gLRP1-a: GCCAAACGAGCATAACTGCC, gLRP1-b: CATTGTGTCCCCA-CACTCGA) were designed using CHOPCHOP and cloned into pLentiGuide-puro (Addgene plasmid #52963). HEK293 cells and HeLa cells stably expressing FLAG–Cas9 were infected with lentivirus harboring pLentiGuide-puro gEXT1 (HEK293), pLentiGuide-puro gLRP1-a, and pLentiGuide-puro gLRP1-b (HeLa), respectively. As a control, HEK293 cells and HeLa cells were infected with lentivirus harboring pLentiGuide-puro gControl (CGCAGTCATTCGATAGGAAT). After 24 h of transduction, cells were cultured with 1 μg/mL puromycin (HEK293 and HeLa) and 100 ng/mL dox (only HEK293) for 7 days; they were then used as KO cell lines.

### Preparation of cell lysate and immunoblotting

Cells were washed with cold phosphate-buffered saline (PBS) and lysed in lysis buffer (1% Triton X-100, 50 mM Tris/HCl, pH 7.5, 1 mM ethylenediaminetetraacetic acid [EDTA], and 150 mM NaCl) supplemented with protease inhibitor cocktail (EDTA-free; Nacalai Tesque) and 1 mM phenylmethanesulfonyl fluoride for 15 min at 4 °C. The lysates were clarified by centrifugation at 20,630 × *g* for 5 min at 4 °C, then mixed with sodium dodecyl sulfate (SDS) sample buffer. Samples were boiled at 95 °C for 5 min prior to SDS–polyacrylamide gel electrophoresis (SDS-PAGE). We separated 10 μg of protein per lane via SDS–PAGE; proteins were then transferred to polyvinylidene difluoride membranes (Millipore). Immunoblotting analysis was performed using the indicated antibodies and immunoreactive proteins were visualized using the ImmunoStar Zeta reagent (Wako).

### Flow cytometry

Cells were detached from dishes with trypsin and EDTA for collection, then passed through a 70-μm cell strainer and resuspended in 5% newborn calf serum plus 1 μg/mL 4′,6-diamidino-2-phenylindole (DAPI) in PBS. Flow cytometry was performed using a CytoFLEX S flow cytometer equipped with NUV 375-nm (DAPI), 488-nm (GFP), and 561-nm (mCherry) lasers (Beckman Coulter). Dead cells were detected by DAPI staining. In each sample, more than 10,000 cells were acquired.

### Immunocytochemistry and fluorescence microscopy

Cells were plated on coverslips and fixed in 3.7% formaldehyde in PBS for 15 min. For immunostaining, fixed cells were permeabilized with 50 μg/mL digitonin in PBS for 5 min, blocked with 10% newborn calf serum in PBS for 30 min, and incubated with primary antibodies for 1 h. After a washing step, the cells were incubated with Alexa Fluor 647-conjugated goat anti-rabbit IgG secondary antibodies (Thermo Fisher Scientific) for 1 h. The stained cells were observed under a confocal laser microscope (FV1000 IX81; Olympus) using a 100 × oil immersion objective lens with a numerical aperture of 1.40.

### Co-immunoprecipitation

Recombinant extracellular chaperones (Fig. 2A: 0.14 μM, Fig. 2E, S4: 0.1 μM) were mixed with protein substrates (e.g., BCL or CA1) that had been incubated at 50 °C for 1 h. For BCL, tissue lysate was centrifuged at 2290×*g* for 2 min to remove debris after heat shock. GFP-nanobody or ALFA tag-nanobody sepharoses were added to the mixture and incubated for 2 h at 4 °C. The sepharoses were washed four times with PBS and then transferred to fresh tubes; subsequently, they were subjected to elution with SDS sample buffer.

### Preparation of blood cell lysate

Blood samples were collected from C57BL/6 mice and centrifuged at 2290×*g* for 2 min to separate plasma and blood cells. The cells were washed with PBS, resuspended in an equal volume of homogenization buffer (20 mM HEPES, pH 7.4, 1 mM EDTA, 1 mM phenylmethanesulfonyl fluoride, and protease inhibitor cocktail), and homogenized using a 1-mL syringe with a 27-G needle. The homogenized cells were centrifuged at 20,620×*g* for 10 min to remove cell debris. The supernatant was used as blood cell lysate (BCL). The concentration of BCL total proteins was determined by the Bradford method; it was typically 136 μg/μL. For mass spectrometry, BCL was cleared by ultra-centrifugation at 572,000×*g* (Hitachi S110AT) for 30 min, followed by immunoprecipitation.

### Protein purification

Purification of secreted protein from conditioned medium was performed as previously described^33^. Briefly, cells expressing α_2_M–RG, RG–α_2_M, α_2_M–ALFA, α_2_M(Δbait)–RG, or Clu-RG were cultured with doxycycline in advanced DMEM medium for 4 days. The conditioned medium was collected and centrifuged at 780×*g* for 20 min at 4 °C to remove dead cells and debris. Secreted proteins were purified from the conditioned medium via Ni–NTA affinity chromatography. Purified proteins were stored in PBS with 10% glycerol at − 80 °C.

Human CA1, CA2, PSMD5, ALDH1A1, GCLM, and ESD were tagged with ALFA tag and 6xHis-tag, then cloned into pRSET-A. The plasmids were introduced into the BL21(DE3) LOBSTR strain of *Escherichia coli*^47^, then induced with 0.1 mM isopropyl β-D-1-thiogalactopyranoside at 18 °C. The cells were disrupted by sonication. After ultracentrifugation, recombinant protein was purified from the soluble fraction using HisPur Cobalt Resin (Thermo Fisher Scientific). Purity was assessed by SDS-PAGE with coomassie brilliant blue staining (Fig. S2).

### Internalization assay

Conditioned advanced DMEM containing secreted α_2_M–RG (in which the extracellular chaperone concentration was diluted to 70 nM with advanced DMEM) was mixed with protein substrates (1.09 μg/μL BCL) (2 μM PSMD5, 0.5 μM ALDH1A1, 1 μM ESD in Figs. 2D, 3, 4B, C, 5B) (2 μM CA1, 2 μM Ca2, 2 μM PSMD5, 2 μM ALDH1A, 2 μM GCLM, 2 μM ESD in Figs. 2C and 5A) then heat-treated at 50 °C or incubated at 4 °C for 1 h. The medium was added to cells in 24-well plates and cultured at 37 °C for 20 h. Cells were then collected and analyzed via flow cytometry or immunoblotting.

### SYPRO Ruby staining

After gel electrophoresis, gels were fixed with 7% acetic acid and 50% methanol solution for 30 min. Fixed gels were incubated with SYPRO Ruby solution (Thermo Fisher Scientific) overnight at room temperature with continuous gentle agitation. The gels were destained during 30 min in 7% acetic acid and 10% methanol solution and then rinsed in deionized water. The gels were visualized using a iBright FL1500 Imaging System (Thermo Fisher Scientific).

### Native-PAGE

Proteins were diluted in sample buffer (100 mM Tris pH8.6, 10% glycerol, 0.0025% bromophenol blue). Native-PAGE was performed on NuPAGE Novex 3 to 8% Tris–acetate gels (Thermo Fisher Scientific) and Tris–glycine running buffer (25 mM Tris, 192 mM glycine, pH8.3) (Fujifilm), with a constant voltage 125 V. Gels were stained using coomassie brilliant blue or SYPRO Ruby staining.

## Supplementary Information

Supplementary Figures.

Supplementary Table 1.

## Data Availability

The datasets used and/or analyzed during the current study are available from the corresponding author on reasonable request.
