# Supplementary material for: Alpha 2-macroglobulin acts as a clearance factor in the lysosomal degradation of extracellular misfolded proteins
Source: Sci Rep. 2023 Mar 28;13:4680. doi: 10.1038/s41598-023-31104-x (PMC10050189; doi:10.1038/s41598-023-31104-x)
Supplement: Supplementary file 1 — Supplementary Figures. [file 41598_2023_31104_MOESM1_ESM.docx]

**
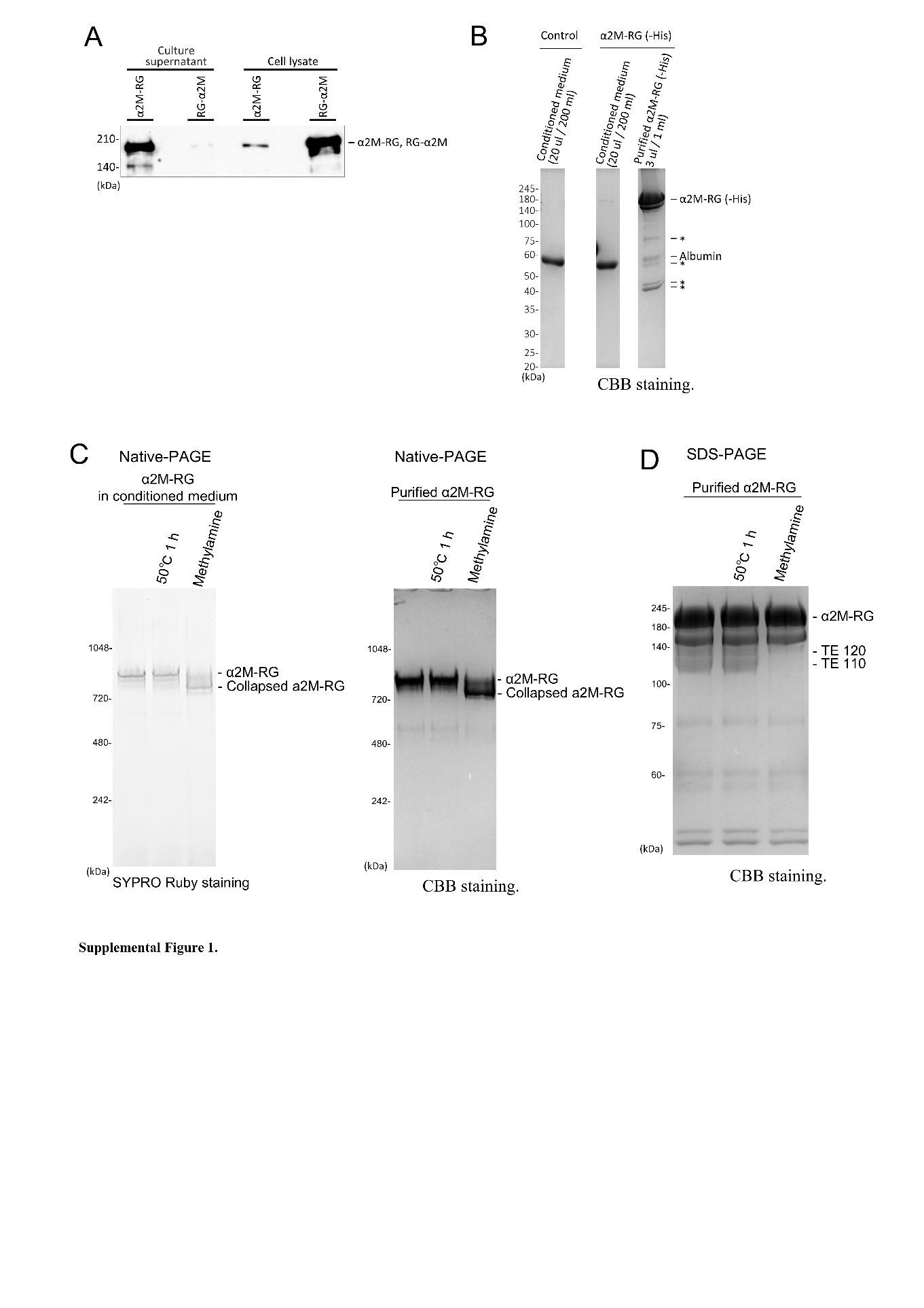
**

**Supplemental Figure 1.** (A) α_2_M–RG–His was secreted in the culture supernatant, whereas RG-α2M was not. α_2_M–RG- or RG–α_2_M-secreting cell lines were cultured; the culture supernatant and cells were each collected and subjected to immunoblotting for assessment of recombinant proteins. (B) α_2_M–RG–His from conditioned medium was purified via Ni-NTA afﬁnity chromatography. Purified α_2_M–RG–His was analyzed by coomassie brilliant blue staining. The asterisk indicates degradation products of α_2_M–RG–His. (C and D) α_2_M–RG–His is a tetramer and native state with an intact thiol ester. α_2_M–RG–His in conditioned medium and purified α_2_M–RG–His were treated with heat shock (50°C for 1 h) or 250 mM methylamine at 37°C overnight. Then, samples were analyzed by native-PAGE and SDS-PAGE. TE110 and TE120 indicate thiol ester dependent heat-fragmentation bands generated as C-terminal autolytic fragment of α_2_M with RFP-GFP (110kDa) and N-terminal fragment (120 kDa).


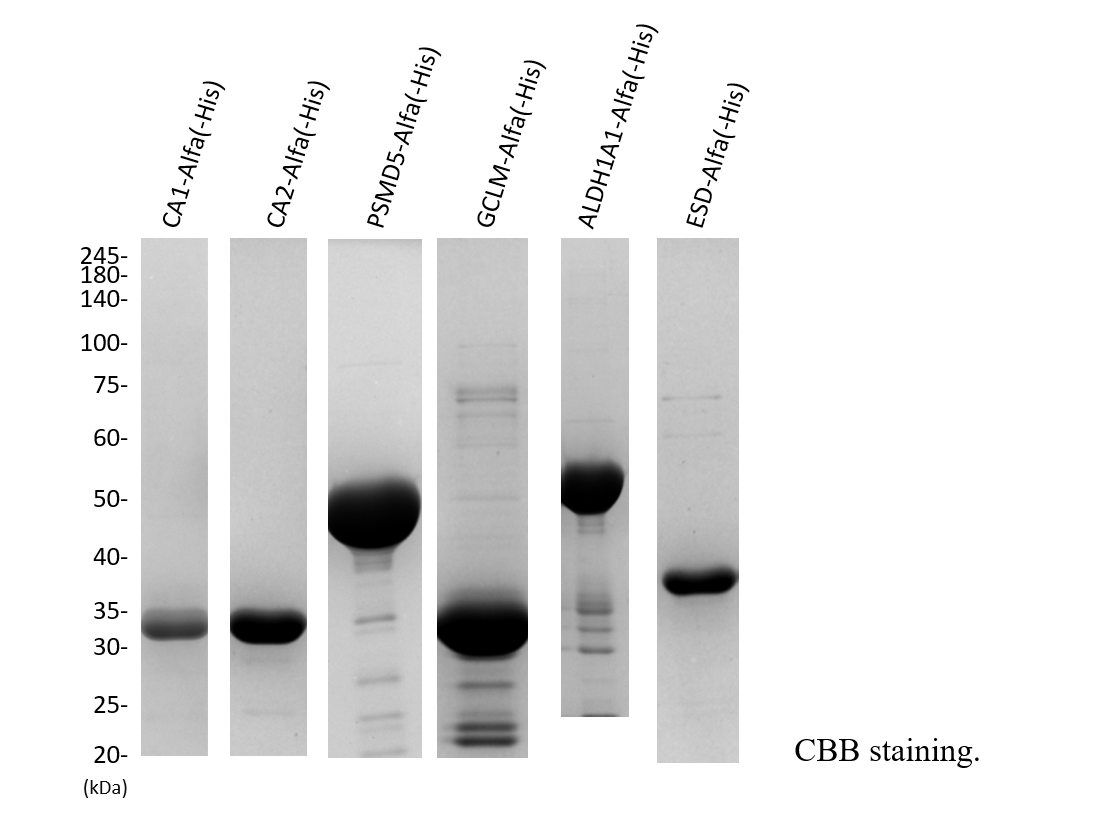


**Supplemental Figure 2.** Purified recombinant human proteins from *Escherichia coli*. were analyzed by coomassie brilliant blue (CBB) staining.


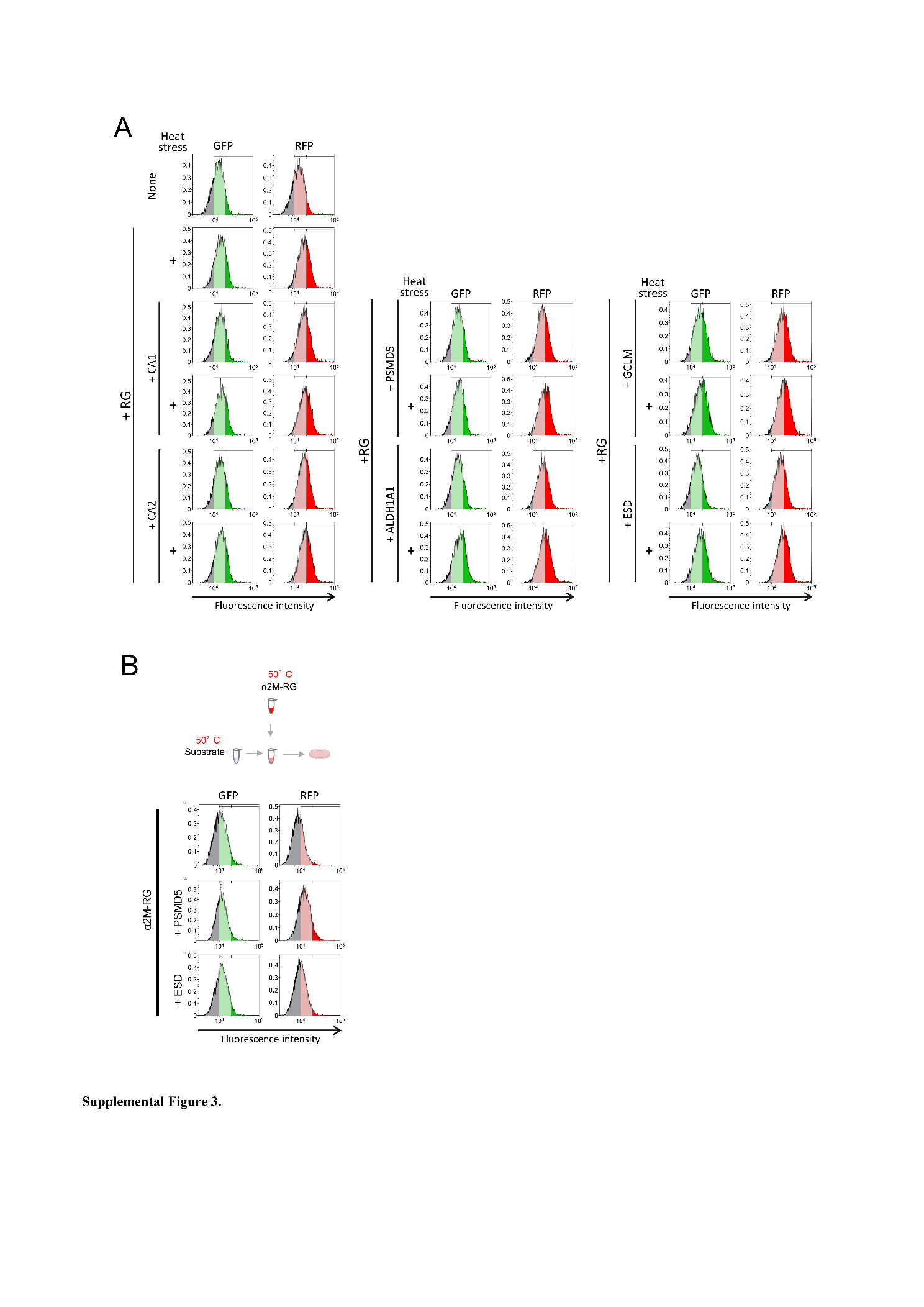


**Supplemental Figure 3.** (A) Heat-stressed proteins did not induce the internalization of RFP–GFP–His. RFP–GFP–His (RG) internalization assays with various substrates (CA1, CA2, PSMD5, ALDH1A1, GCLM, or ESD). RG and substrates were heat-shocked in serum-free medium at 50°C for 1 h. HeLa cells were cultured in medium for 17 h at 37ۥ°C, then analyzed using flow cytometry. (B) Heat shock to α_2_M alone does not stimulate internalization of α_2_M. α_2_M–RG or substrate (PSMD5 or ESD) was pre-heated (50°C 1 h) alone, then mixed with together. HeLa cells were cultured in the medium for 17 h at 37°C, then analyzed using flow cytometry (n = 1).


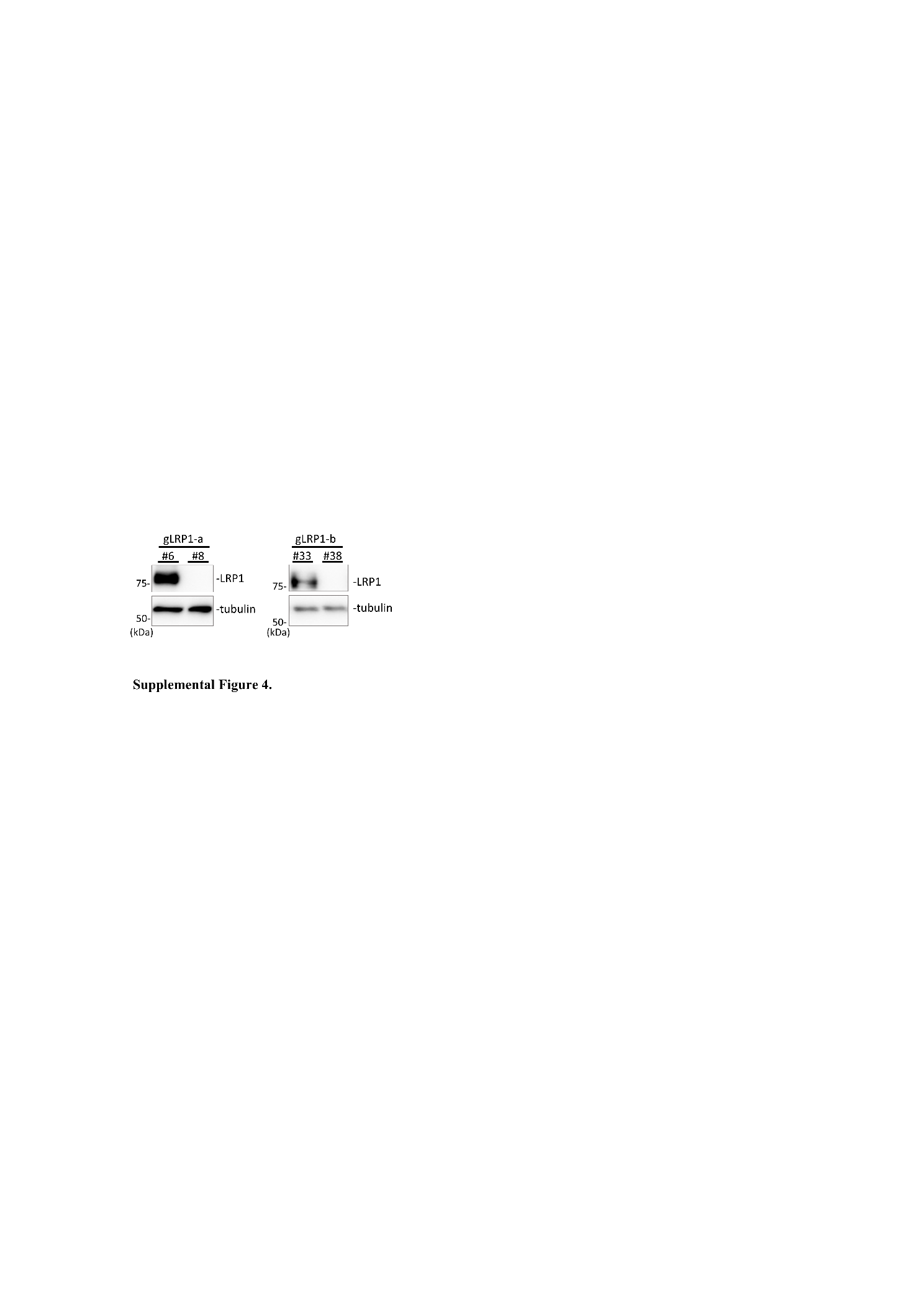


**Supplemental Figure 4.** Among gLRP1-transfected cells, LRP1 KO cells were selected via single-cell cloning. LRP1 KO cell lines were generated using Cas9 and one of two gRNAs (gLRP1-a or gLRP1-b). These cells were distributed into a single cell line. LRP1 expression was not detected in gLRP1 #8 or gLRP1 #38.


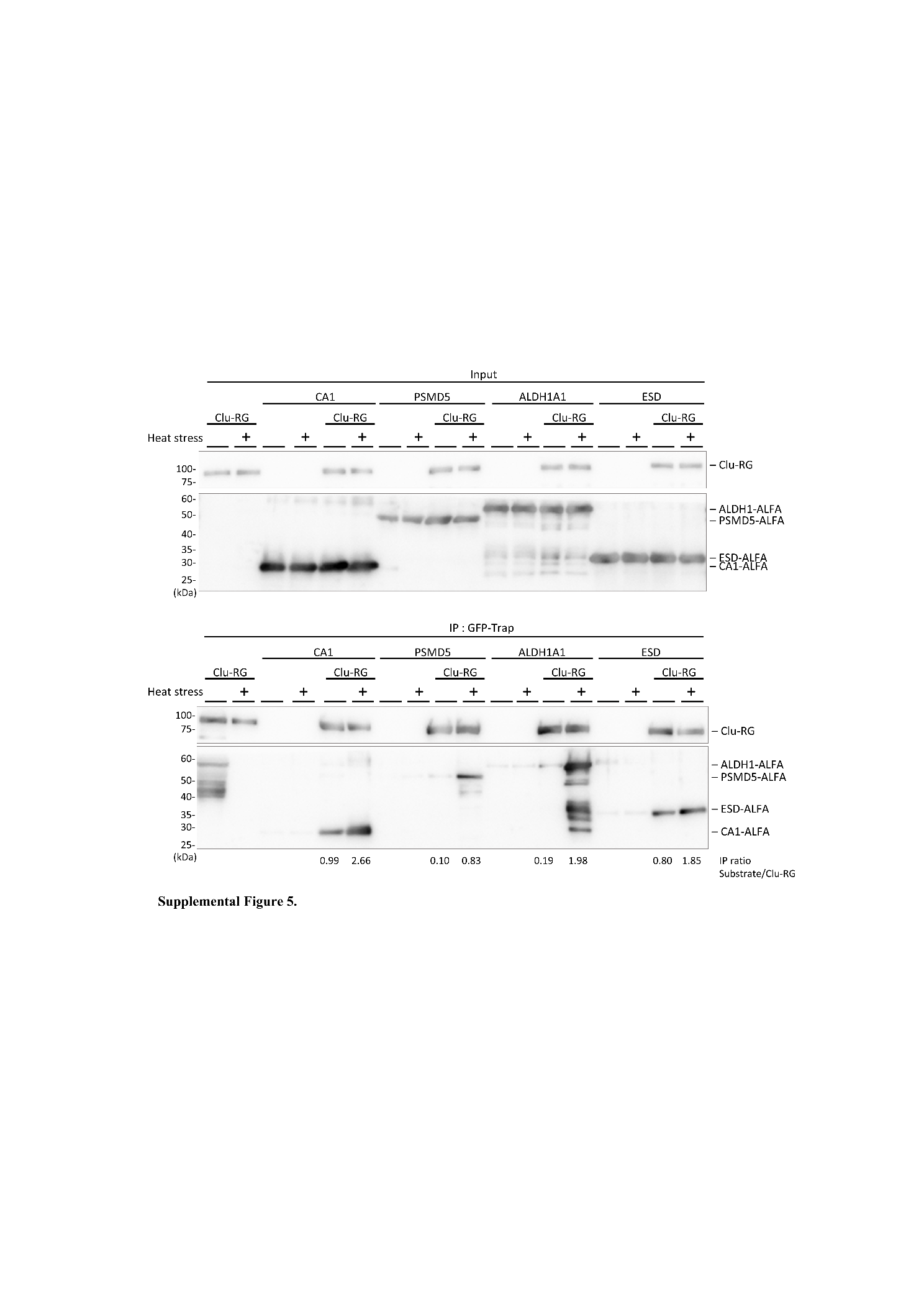


**Supplemental Figure 5.** Clusterin interacts directly with substrates. CA1, PSMD5, ALDH1A1, or ESD was mixed with or without Clu-RG, then pre-incubated at 4°C or 50°C (heat stress) for 1 h. Samples were subjected to co-immunoprecipitation with anti-GFP sepharose. IP ratios were calculated by the band intensity of the substrate divided by the band intensity of the Clu-RG.


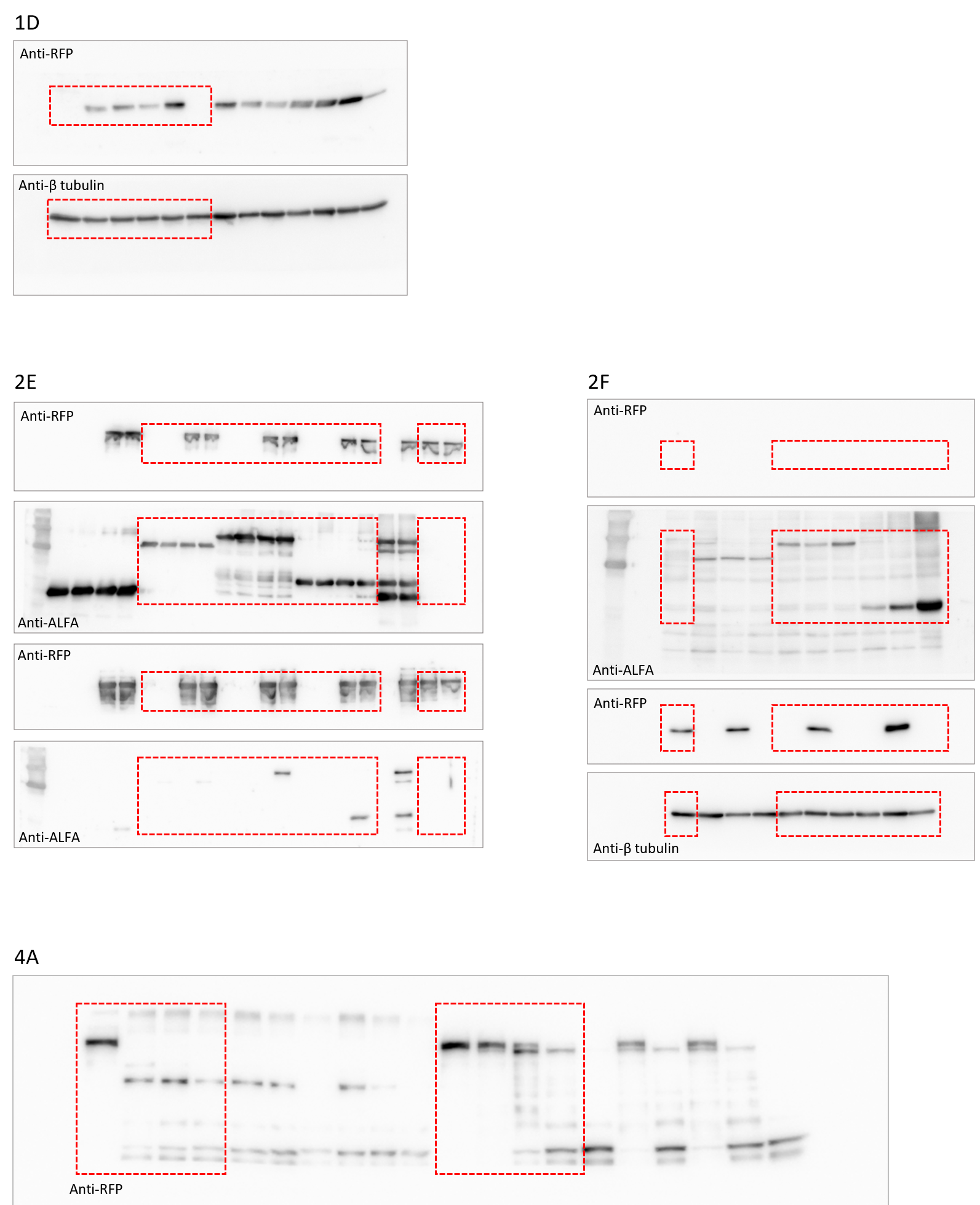


**Supplemental Figure 6.** Full-length blots for analyses shown in Figs. 1D, 2E, 2F, and 4A.


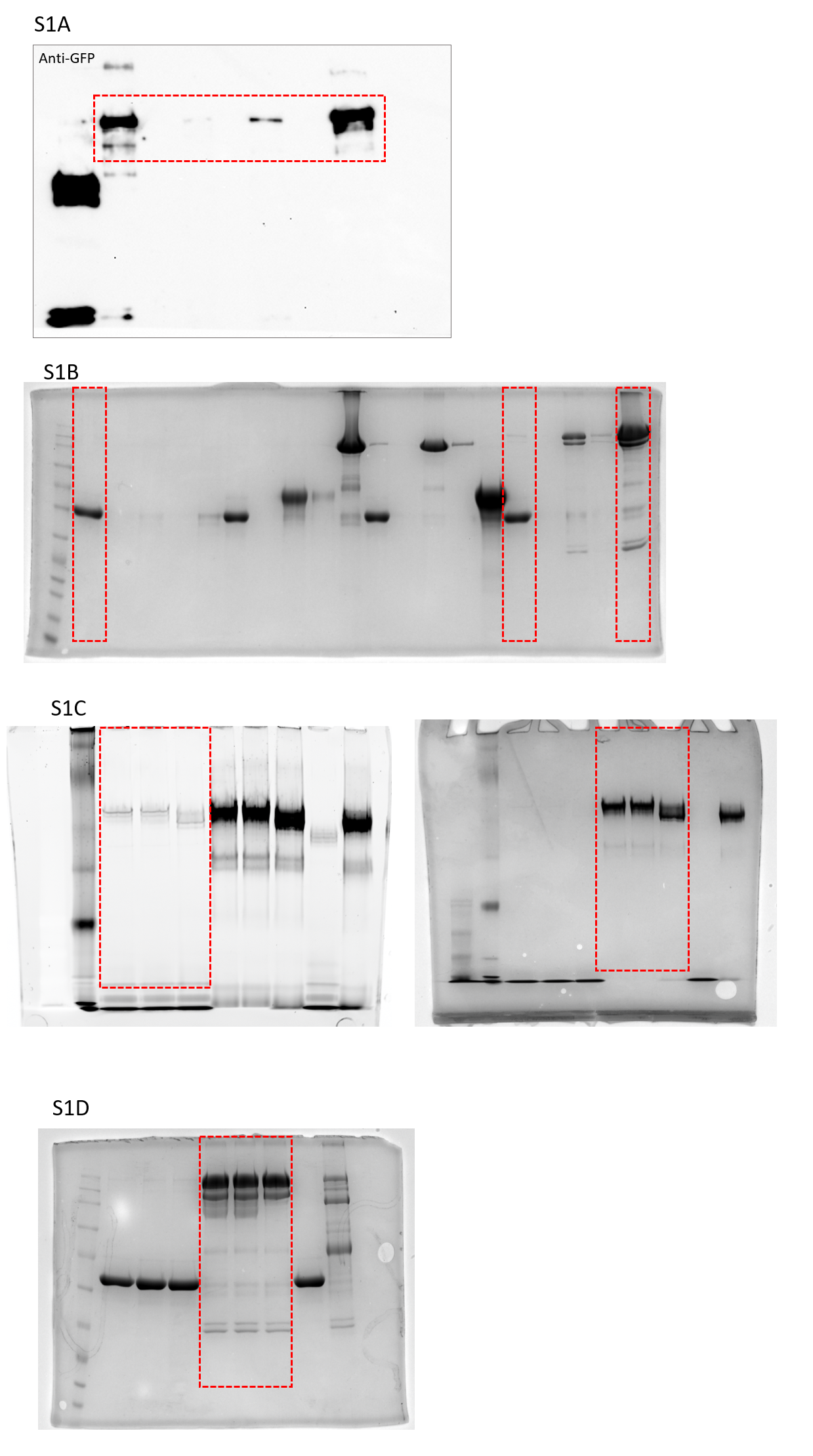


**Supplemental Figure 7.** Full-length blot and gels for analyses shown in Fig. S1.


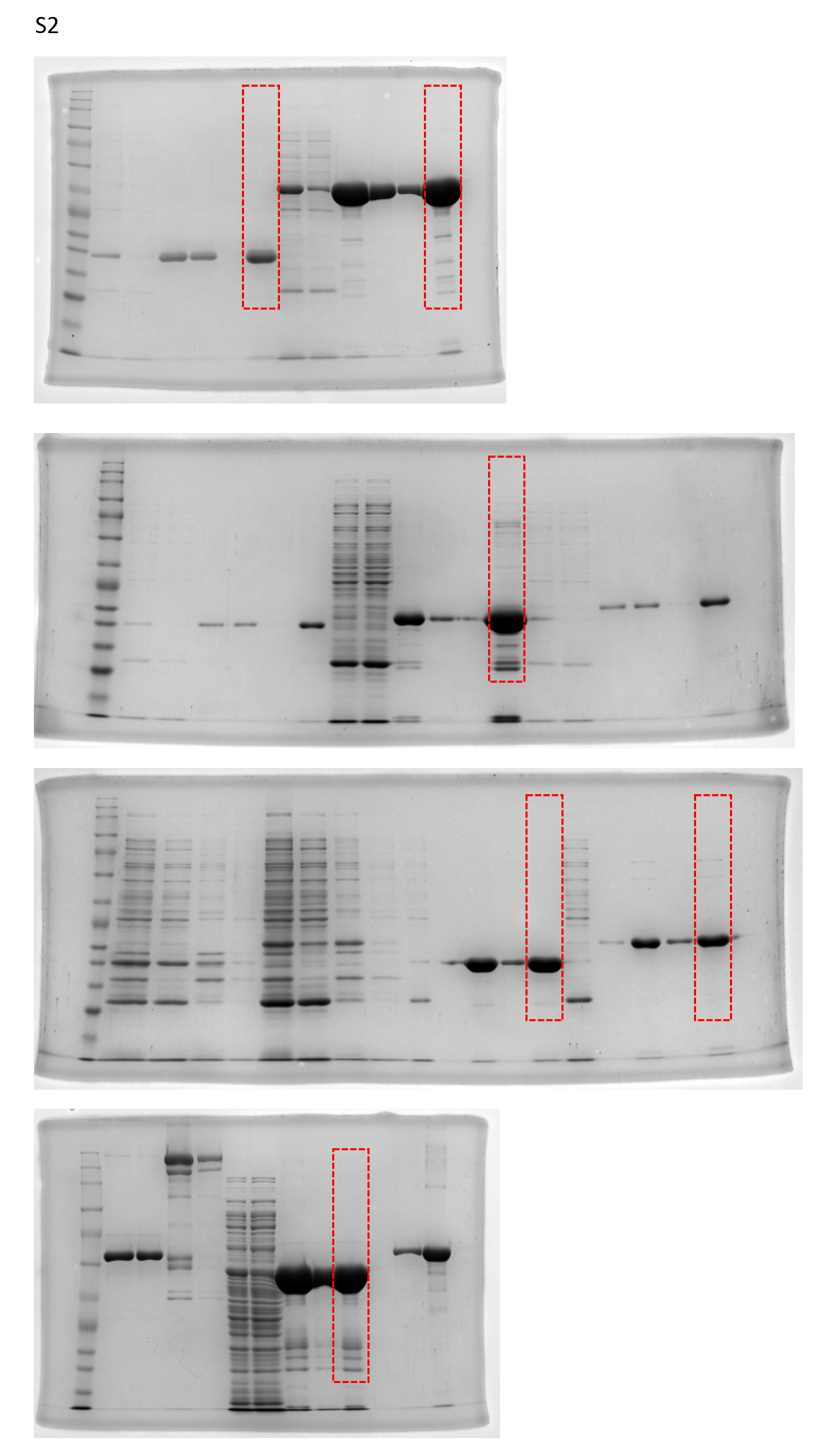


**Supplemental Figure 8.** Full-length gelss for analyses shown in Fig. S2.


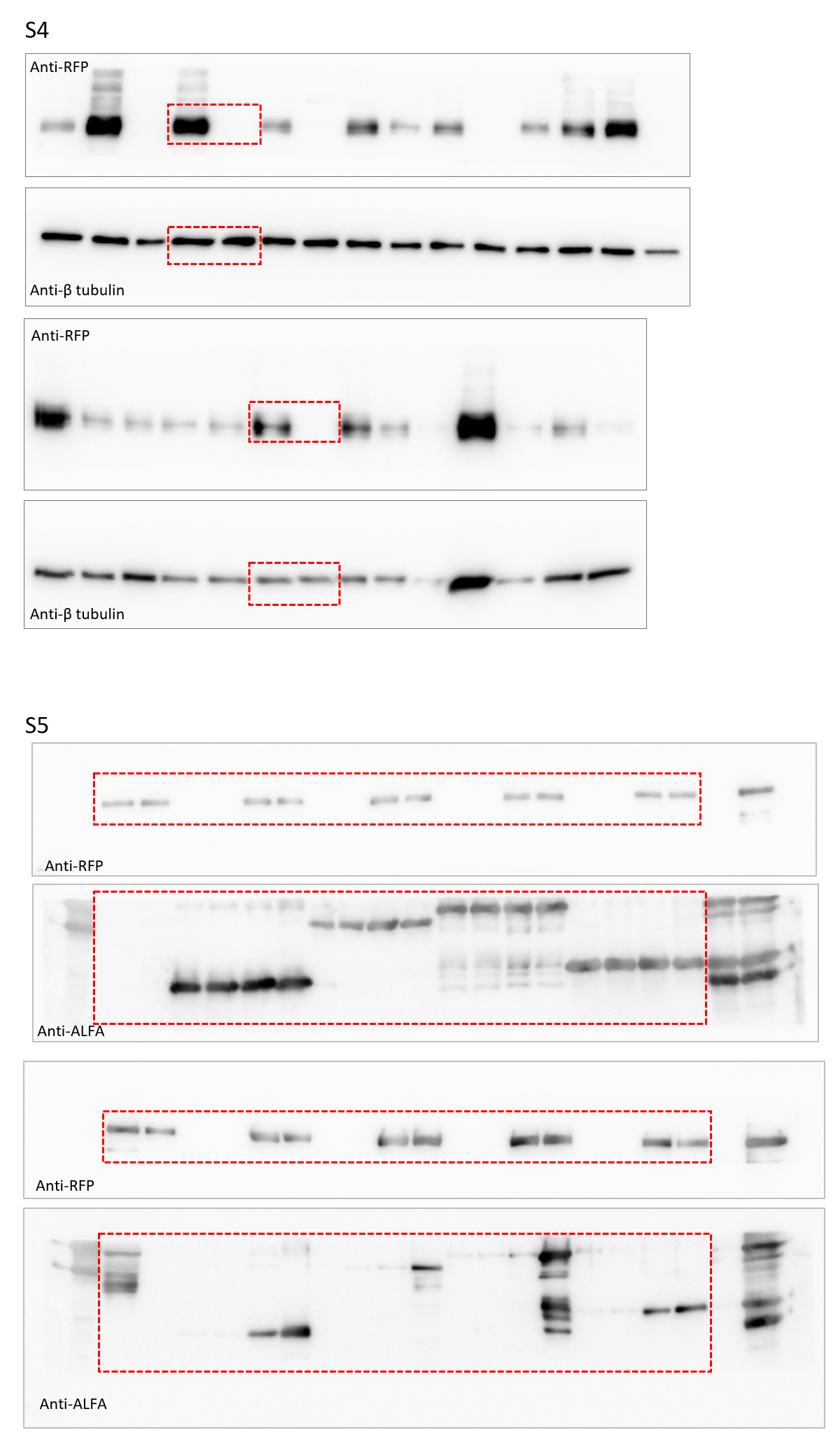


**Supplemental Figure 9.** Full-length blots for analyses shown in Figs. S4, S5.
